# Pancreatic Intraductal Papillary Mucinous Neoplasm With Invasive Carcinoma Concomitant With Ampullary Neuroendocrine Tumor: A Case Report

**DOI:** 10.1002/ccr3.71309

**Published:** 2025-10-31

**Authors:** Jingcheng Zhang, Jianhao Huang, Xiaodong He, Mengqing Sun, Xianlin Han

**Affiliations:** ^1^ Department of General Surgery, Peking Union Medical College Hospital Chinese Academy of Medical Sciences and Peking Union Medical College Beijing China

**Keywords:** ampullary neuroendocrine tumor, lymph node metastasis, multiple primary tumors, pancreatic intraductal papillary mucinous neoplasm

## Abstract

This case reports a rare co‐occurrence of pancreatic intraductal papillary mucinous neoplasm with invasive carcinoma and ampullary neuroendocrine tumor. Laparoscopic pancreaticoduodenectomy was done. No recurrence/metastasis in 12‐month follow‐up, though both tumors had lymph node metastasis, warranting attention to timely biopsy.

## 1 Introduction

The coexistence of pancreatic intraductal papillary mucinous neoplasm (IPMN) and neuroendocrine tumor in a patient is not merely a coincidence. Although the reported cases are limited, it has received increasing attention in recent years. IPMN with pancreatic neuroendocrine tumor (NET) has been reported in some cases, but IPMN with ampullary neuroendocrine tumor has not been reported. This article reports a case of a pancreatic head mass patient, whose postoperative pathology suggested IPMN with invasive carcinoma, combined with a neuroendocrine tumor in the ampulla. Laparoscopic pancreaticoduodenectomy was performed and adjuvant chemotherapy was suggested. During the 12‐month follow‐up, the patient recovered well without recurrence and metastasis. Both tumors were associated with lymph node metastasis, making it a rare but noteworthy case.

## 2 Case Presentation

### 2.1 Present Illness

This is a 52‐year‐old male who presented with left upper‐quadrant colicky abdominal pain for 4 months, rated 8 on the NRS, starting 30 min post‐meals, slightly relieved by knee‐chest position, with no fever, vomiting, diarrhea, or hematochezia. Since illness onset, appetite was normal, mood and sleep good, with a 10 kg weight loss.

### 2.2 Past Medical History, Family History, and Physical Examination Findings

Past medical history included 1‐year diabetes, treated with metformin 0.5 g bid, no regular blood glucose monitoring. The patient had a history of smoking for 20 years, smoking 20 pieces of cigarettes per day. The patient denied any family history of similar conditions, including psychiatric disorders, neoplasms, or hereditary diseases. Physical examination: No jaundice was observed in the skin or mucous membranes. Visual inspection revealed a flat abdomen with no visible gastrointestinal patterns, peristaltic waves, masses, or dilated abdominal wall veins. Palpation demonstrated a soft abdomen without significant tenderness, rebound tenderness, or muscle guarding. No hepatic tenderness or percussion pain was detected. Murphy's sign and Courvoisier's sign were negative. Abdominal percussion revealed tympany with no shifting dullness. Bowel sounds were approximately 3 per minute.

### 2.3 Imaging Studies

Abdominal enhanced CT showed a 3.7 × 2.5 cm mass between the pancreatic head and duodenum with mild peripheral enhancement after contrast administration, suggesting pancreatic cancer, mass‐forming pancreatitis, or neuroendocrine tumor. Meanwhile, the superior mesenteric vein was invaded.

## 3 Differential Diagnosis, Investigations, and Treatment

### 3.1 Differential Diagnosis

#### 3.1.1 Pancreatic Cancer

Early‐stage pancreatic cancer is often asymptomatic. Advanced cases may present with jaundice, back pain, and weight loss. CA19‐9 is frequently elevated. On CT, the tumor typically appears isodense or mixed isodense—hypodense with normal pancreatic tissue on plain scan. As it is a hypovascular tumor, there is minimal, slow, or uneven enhancement in the early phase of contrast‐enhanced CT. This patient has abdominal symptoms, weight loss, and the enhanced CT showed superior mesenteric vein (SMV) invaded. The diagnosis is highly suspected but requires surgical pathology for confirmation.

#### 3.1.2 Pancreatic Neuroendocrine Tumor (PNET)

Most PNETs are nonfunctional and don't cause hormone‐secretion syndromes. In advanced stages, they may present with symptoms of local compression and metastases. Diagnosis can be made using radiolabeled somatostatin analogs for diagnostic imaging. Multiphasic CT or MRI helps determine the extent of disease spread and the potential primary site. PNETs usually show significant enhancement in the early arterial phase. Given the patient's unremarkable enhancement of the mass on contrast‐enhanced CT, this diagnosis is less likely and needs postoperative pathology for clarification.

#### 3.1.3 Intraductal Papillary Mucinous Neoplasm (IPMN)

In most cases, excessive mucin secretion leads to cystic dilation of the involved pancreatic duct, while in a minority, local or diffuse papillary proliferation causes ductal dilation. It is slightly more common in males, with an average age of onset around 65 years. Acute or chronic pancreatitis symptoms are most frequent, and it can also be incidentally detected during physical examinations. Based on the patient's contrast‐enhanced CT findings and biopsy results, this diagnosis is not currently considered but requires postoperative pathology to rule it out.

### 3.2 Further Examination and Treatment

**FIGURE 1:**
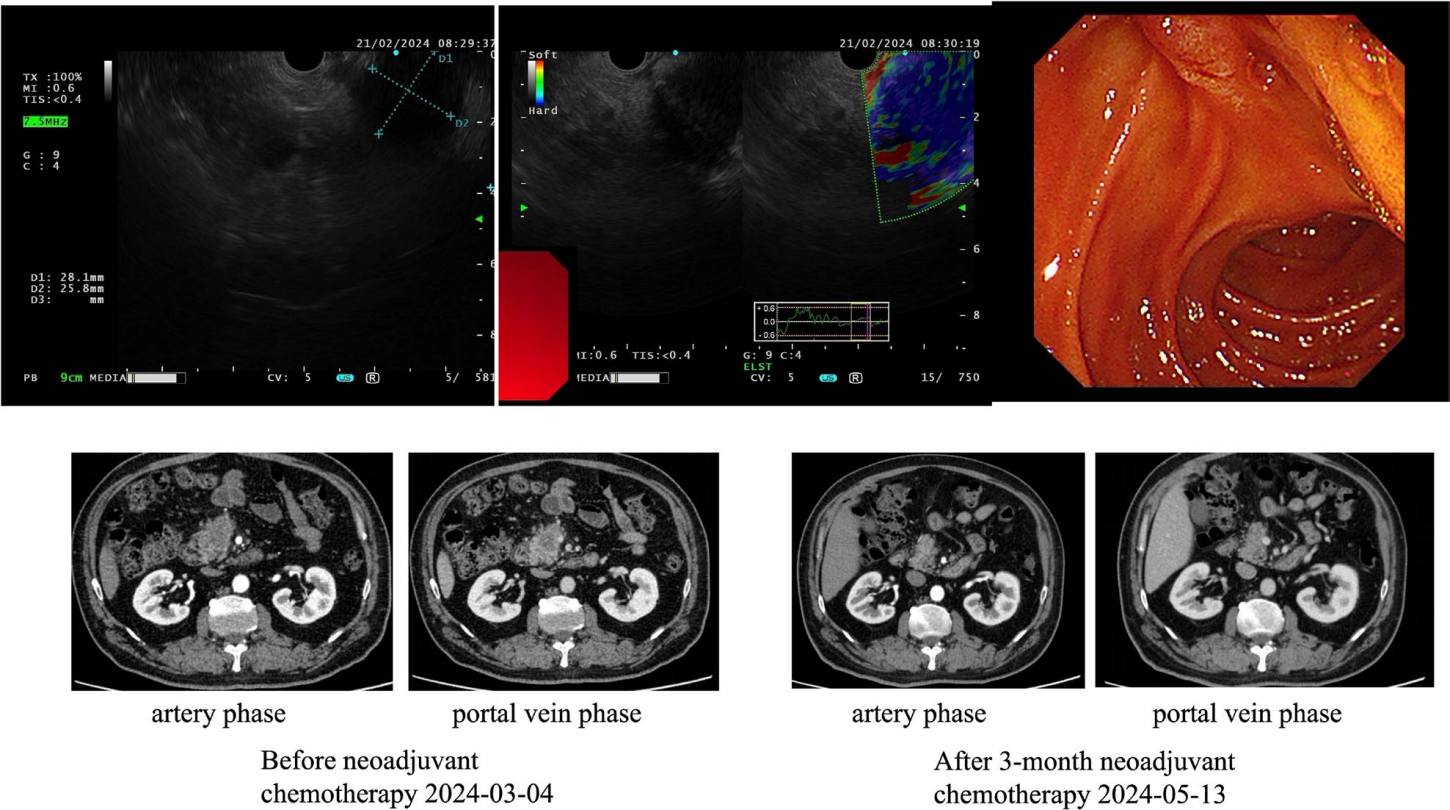
FNB‐EUS results and enhanced CT results. The endoscopic ultrasonography showed a hard mass in the pancreatic head. The enhanced CT scan indicated that after three cycles of chemotherapy, the tumor had significantly shrunk, and the involvement of the superior mesenteric vein (SMV) had decreased compared to the previous state.

Endoscopic ultrasound (EUS) was performed and showed a hypoechoic mass in the pancreatic head of approximately 2.8 × 2.5 cm (Figure 1). Pathological analysis of the fine needle biopsy (FNB) indicated adenocarcinoma.

Due to the close relationship between the superior mesenteric vein and the lesion, as indicated by enhanced CT, local advanced pancreatic cancer was suspected, and neoadjuvant chemotherapy was recommended.

**FIGURE 2:**
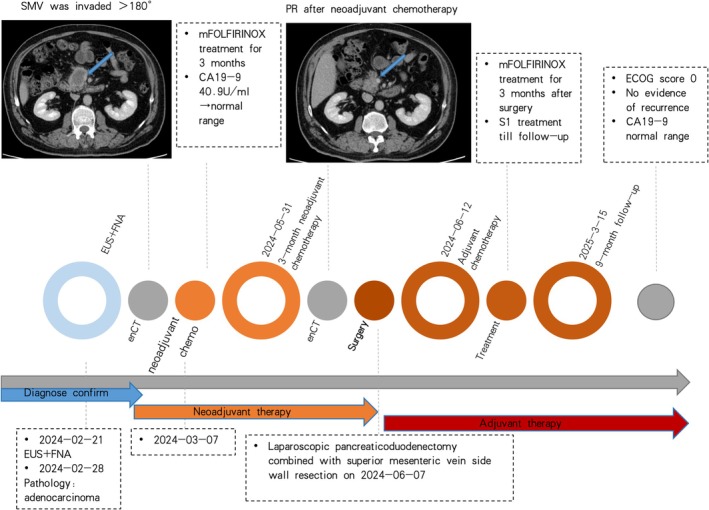
Timeline of treatment and clinical imaging evaluation result.

Administration of the modified FOLFIRINOX regimen (Oxaliplatin 85 mg/m^2^ on day 1 + Irinotecan 180 mg/m^2^ on day 1 + Leucovorin Calcium 400 mg/m^2^ on day 1 + 5‐Fluorouracil 2400 mg/m^2^ via continuous intravenous infusion over 46 h, every 2 weeks). After completing three cycles of mFOLFIRINOX regimen chemotherapy, a follow‐up enhanced CT of the abdomen showed a reduction in the size of the pancreatic head mass and decreased vascular invasion. Subsequently, laparoscopic pancreaticoduodenectomy was performed in June 2024. (Figure 2).

### 3.3 Pathology

Postoperative pathology revealed intraductal papillary mucinous neoplasm (IPMN) of the pancreatic duct with invasive carcinoma (2.5 × 2 × 1 cm, moderately to poorly differentiated adenocarcinoma, some of which are mucinous adenocarcinoma), involving surrounding pancreatic fat tissue, accompanied by fibrous tissue proliferation, with partial tumor response to treatment. TNM stage is ypT2N1M0. In the ampulla, a neuroendocrine tumor (NET, G2) was also identified. Lymph node metastasis was present (5 out of 37), with two lymph nodes showing metastatic adenocarcinoma and three lymph nodes showing metastatic neuroendocrine tumor. Immunohistochemistry results: B10: IMP3 (+), P53 (+), Ki‐67 (index 80%), MUC1 (+), MUC2 (partially +), MUC5AC (+), MUC6 (partially +), DPC4 (+), INSM‐1 (−), CgA (−).

B11: AE1/AE3 (+), CgA (partially +), SSTR2 (3+; strong positive), Syn (+), INSM‐1 (+), Ki‐67 (index 3%). B17: CgA (partially weak +), INSM‐1 (+), Syn (+), Ki‐67 (index 3%), AE1/AE3 (+). B19: INSM‐1 (+). B20: INSM‐1 (+).

Notes:

“+” = positive; “−” = negative; “partially +” = focal/partial positivity; “3+” = strong positivity; “index X%” = labeling index (proportion of positive cells).

**FIGURE 3:**
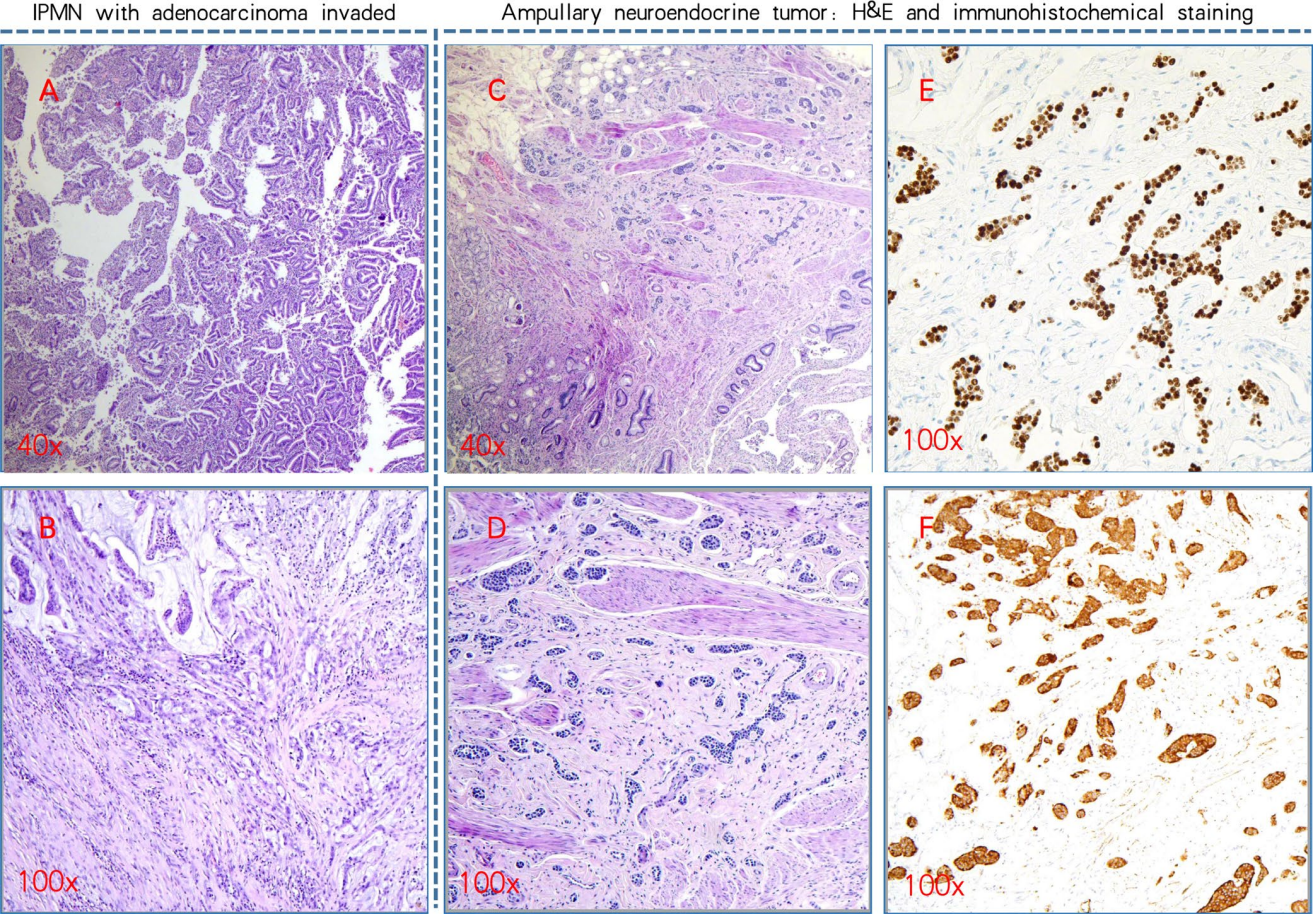
Pathological images of IPMN with invasive carcinoma and ampullary neuroendocrine tumor (G2). (A) Demonstrates the histopathology of the patient's intraductal papillary mucinous neoplasm (IPMN) (40× magnification). (B) Highlights the invasive carcinoma region identified within the IPMN (100× magnification). (C–F) Illustrate the pathology of a neuroendocrine tumor (NET) located at the patient's ampulla, with (E) and (F) showcasing immunohistochemical staining results: Synaptophysin (Syn) positivity (+) and insulinoma‐associated protein 1 (INSM‐1) positivity (+).

Markers like AE1/AE3 are pan‐cytokeratin antibodies, and SSTR2 (3+) indicates strong membrane staining (Figure 3).

## 4 Outcome and Follow‐Up

The patient demonstrated favorable postoperative recovery. One month after surgery, the modified FOLFIRINOX (mFOLFIRINOX) regimen was reinitiated and continued for 3 months. Following reassessment by the medical oncology team, the patient transitioned to oral S‐1 monotherapy for disease control. At the time of follow‐up, the patient had resumed a regular diet, with no weight loss or decline in functional status, and continued oral S‐1 administration. Surveillance every 2 months showed no evidence of recurrence on tumor markers or CT imaging. However, the 1‐year follow‐up period is relatively short, and closer monitoring is required. Both tumor types carry a risk of late recurrence; therefore, a minimum 5‐year monitoring protocol is recommended, with alternating contrast‐enhanced CT/MRI every 3–6 months.

## 5 Discussion

The coexistence of pancreatic intraductal papillary mucinous neoplasm (IPMN) and neuroendocrine tumor in a patient is not merely a coincidence. IPMN with pancreatic neuroendocrine tumor has been reported in some cases, but IPMN with ampullary neuroendocrine tumor has not been reported yet.

Intraductal papillary mucinous tumors (IPMNs) of the pancreas are mucus‐producing ductal exocrine lesions. They account for 1%–3% of pancreatic exocrine tumors and 20% of pancreatic cyst tumors [1]. Neuroendocrine tumors of the ampulla are much less common and have only been reported in a few cases [2, 3]. Intraductal papillary mucinous tumors are most frequently associated with concurrent pancreatic ductal adenocarcinoma (PDAC), which may be present in 2%–10% of resected IPMNs [4]. In this case, the patient came to the clinic with a hypoechoic mass in the pancreatic head. FNB indicated adenocarcinoma, and the pathology confirmed IPMN with invasive carcinoma.

Among other pancreatic lesions that may be associated with IPMN, PNET is the second most common. It has been suggested that cells in the tumor process can differentiate from one cell type to another, or that two cell types may come from the same tumor precursor [5, 6].

The literature reports only about 150 cases of NETs originating from the ampulla of Vater, most of which are nonfunctional and do not present with carcinoid syndrome [7]. For ampullary NETs, radical surgical resection is currently recommended due to the fact that approximately 50% of cases involve lymph node involvement or metastasis. Patients can also use functional imaging to find lymph node metastasis according to recent studies [8, 9]. In this case, the patient had lymph node metastasis of ampullary NET and cancerous IPMN at the same time, and there may be a mechanism of synergistic metastasis between tumors.

The coexistence of pancreatic IPMN and neuroendocrine tumor is a noteworthy condition. They can present as concurrent tumors or mixed endocrine‐exocrine tumors. The pathogenesis of this condition is not clear. For the coexistence of nonfunctional small NETs, the preoperative diagnostic rate is low, and most patients are incidentally discovered on postoperative pathology. Preoperative EUS can be helpful in improving diagnostic rates. Due to the potential for metastasis in the NETs associated with IPMN, there is a risk of misdiagnosis. We believe that a careful preoperative imaging evaluation, especially EUS‐FNB, may be necessary.

## Author Contributions

**Jingcheng Zhang:** formal analysis, investigation, writing – original draft. **Jianhao Huang:** data curation, writing – original draft. **Xiaodong He:** supervision, writing – review and editing. **Mengqing Sun:** investigation, methodology, writing – review and editing. **Xianlin Han:** conceptualization, resources, supervision, writing – review and editing.

## Ethics Statement

This study was conducted in strict accordance with the ethical principles outlined in the Declaration of Helsinki and applicable local/national regulations. Written informed consent was obtained from the patient or his legally authorized representatives prior to enrollment, as required by the journal's policy on patient consent. A signed copy of the consent form has been retained in the study records.

## Conflicts of Interest

The authors declare no conflicts of interest.

## Data Availability

The data are available from the corresponding author on reasonable request.
